# Endoscopic Therapy of Small Bowel Polyps by Single-Balloon Enteroscopy in Patients with Peutz–Jeghers Syndrome

**DOI:** 10.1155/2022/7849055

**Published:** 2022-02-01

**Authors:** Zhongsheng Cao, Weili Jin, Xueping Wu, Wensheng Pan

**Affiliations:** ^1^Department of Gastroenterology, Zhejiang Provincial People's Hospital, People's Hospital of Hangzhou Medical College, Hangzhou, Zhejiang, China; ^2^BengBu Medical College, Bengbu, Anhui, China; ^3^Department of Gastroenterology, People's Hospital of Nanxun District, Huzhou, Zhejiang, China

## Abstract

**Background:**

Little is known about the efficacy and safety of single-balloon enteroscopy (SBE) in patients with Peutz−Jeghers syndrome (PJS). The aim of this study was to assess the efficacy and safety of SBE for the treatment of small bowel polyps in patients with PJS.

**Methods:**

We conducted a single-center observational study, which included all patients diagnosed with PJS who underwent SBE for polypectomy between January 2018 and March 2021. Complete treatment was defined as the absence of polyps ≥10 mm after SBE resection. The clinical records were retrospectively reviewed.

**Results:**

102 patients (including 40 men and 62 women) with a mean age of 28.7 years (range 13–55 y) were enrolled in our study. The intubation depth via the oral approach of patients with a history of laparotomy was significantly shorter than that of the patients without a history of laparotomy ([241.6 ± 64.2] cm vs [280.9 ± 40.2] cm, *P*=0.008). The maximum size of the resected polyps via anus during the second hospitalization was significantly smaller than that during the first hospitalization ([2.25 ± 1.29] cm vs [4.26 ± 3.51] cm, *P*=0.032). For patients with total enteroscopy, the complete treatment rate was 98% (49/50). For patients without total enteroscopy, all polyps larger than 10 mm in the examined segment of small bowel were resected successfully. Complications occurred in 10 of 129 hospitalizations (delayed bleeding in 4, perforation in 3, and acute pancreatitis in 3).

**Conclusions:**

SBE is effective and safe for resection of small bowel polyps in patients with PJS.

## 1. Introduction

Peutz−Jeghers syndrome (PJS) is a rare autosomal dominant hereditary disorder characterized by mucocutaneous melanin pigmentation and multiple gastrointestinal (GI) hamartomatous polyps [1]. Hamartomatous polyps can be detected in the small bowel, most commonly in the jejunum [2]. These hamartomatous polyps in the small bowel can lead to bleeding, intussusception, and obstruction [3, 4]. Consequently, patients often undergo multiple laparotomies with intestinal resection, which can ultimately result in short-bowel syndrome and/or severe adhesions [4, 5]. It is well acknowledged that large polyps (10–15 mm) or symptomatic or rapidly growing polyps should be removed [2, 6, 7]. In addition, the risks of gastrointestinal and non-gastrointestinal malignancies significantly increase in patients with PJS [8, 9].

Double-balloon enteroscopy (DBE) was first introduced by Yamamoto et al. in 2001. The major advantage of DBE is that a wide variety of therapeutic interventions can be performed during the examination procedure [10–12]. Over the last decade, DBE has been reported to be useful for the treatment of small bowel polyps in patients with PJS [13–16]. The data on the usefulness of DBE for the treatment of polyps were focused on adults. However, the diagnosis and treatment procedures in children (aged 0–17 years old) with PJS may be difficult. The key reasons are as follows: (1) narrow intestinal lumen, (2) thinner intestinal wall, and (3) sharper angle of enteroscopy [17]. The evidence in regard to the role of enteroscopy in children with PJS is limited. Single-balloon enteroscopy (SBE) was first introduced in 2007. The main advantages of SBE compared with DBE are ease of setup, shorter procedure time, and lower operative cost [18, 19].

The aim of this study was to evaluate the efficacy and safety of SBE in patients with PJS. In detail, the aims were as follows: (1) to evaluate the efficacy and safety of SBE in patients with PJS; (2) to evaluate the influence of laparotomy on the SBE procedure; and (3) to evaluate the efficacy and safety of SBE in children.

## 2. Patients and Methods

### 2.1. Patients

We conducted an observational retrospective study which included all patients with PJS from Zhejiang Provincial People's Hospital (People's Hospital of Hangzhou Medical College, Hangzhou, China), a tertiary-care referral center, who had undergone SBE for polypectomy between January 2018 and March 2021. Medical records of all the included patients were retrospectively reviewed. The following data were collected: age, gender, history of laparotomy, the method of SBE insertion, number of SBE procedures performed, maximum size and number of the resected polyps, and complications. A clinical diagnosis of PJS can be made when any one of the following was present: (1) two or more histologically confirmed PJS polyps; (2) any number of PJS polyps detected in one individual who has characteristic mucocutaneous pigmentation or a family history of PJS in close relatives; and (3) characteristic mucocutaneous pigmentation in an individual who has a family history of PJS in close relatives [20]. The study protocol was approved by the Ethics Committee of Zhejiang Provincial People's Hospital (Approval Number 2021QT244). The study protocol was reviewed and approved by the Institutional Review Board and Ethics Committee of Zhejiang Provincial People's Hospital (Approval Number 2021QT244).

### 2.2. SBE Procedure

Before SBE procedures, patients were advised to abdominal ultrasound and CT enterography (CTE) for primary evaluation of small bowel. All the SBE procedures were performed by experienced endoscopists using the SIF-Q260 (Olympus, Japan) with a 200 cm working length and 2.8 mm working channel. In general, both anal and oral SBE procedures were performed. Unless it was clear that no polyps were present in the ileum, the anal approach was performed first because large polyps removed via the oral route may pile up on remaining distal polyps and cause obstruction or intussusception. Patients were advised to fluid diet the day before SBE operation, and polyethylene glycol-electrolyte powder was used for intestinal cleaning. Because the SBE procedure time was long, all examinations were carried out with patients under general anesthesia with endotracheal intubation.

**Figure 1:**
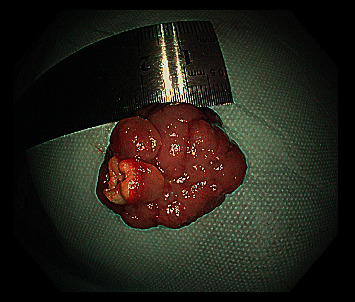
A retrieved polyp was measured with a ruler to determine the greatest dimension.

**Figure 2:**
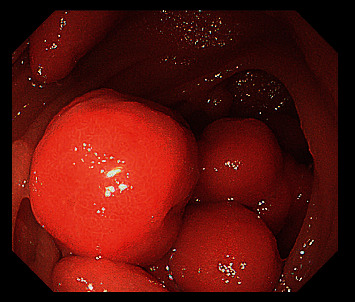
Multiple polyps observed under SBE.

**Figure 3:**
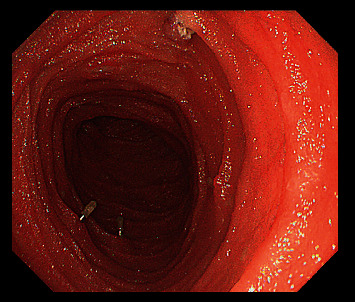
Endoclips were used at the deepest position for mark.

Polyps were resected during withdrawing endoscope to avoid bleeding and perforation at the wound after polypectomy. A polypectomy snare (NOE342216-G, ENDO-FLEX (Suzhou) Co. Ltd) was used to resect a polyp as one block or multiblock according to the size and pattern of the base. The polyp size was estimated according to the width of the biopsy forceps or the diameter of the polypectomy snare. The retrieved polyps were measured with a ruler to determine the greatest dimension (Figure 1). Polyp resection was performed for polyps larger than 10 mm. However, if many polyps were found, polyps of over 20 mm in diameter were given priority for resection to prevent intussusception [14] (Figure 2). Complete treatment was defined as the absence of polyps ≥10 mm after SBE resection. If all of the large polyps could not be removed in one hospitalization, next SBE treatment was repeated within 6 months. Usually, endoclips (ROCC-D-26-230-C, Micro-Tech (Nanjing) Co. Ltd) were used to close the wound to prevent delayed bleeding and perforation. Only large and/or irregularly shaped polyps were retrieved for pathological examination. During the first intubation of SBE, endoclips were used at the deepest position for mark (Figure 3). Insertion depth was measured by accumulation of net advancement of each push-and-pull maneuver as described by Rondonotti et al. [21]. Complications related to enteroscopy such as bleeding, perforation, and acute pancreatitis were noted. Complications were classified as intraprocedural, early (within 24 hours), or delayed (2–30 days) [15]. All complications were obtained from hospitalization records or outpatient assessment.

### 2.3. Statistical Analysis

Descriptive statistics were calculated for the patients' characteristics and SBE parameters and were presented as medians, means, and ranges. Continuous variables were expressed as mean (standard deviation) or median (range). Categorical variables were presented as numbers and percentages or rates. Differences in numerical variables were analyzed by Student's *t*-test or the Mann–Whitney *U* test. However, differences in categorical variables were analyzed by the *χ*^2^ test or Fisher's exact test. A two-sided *P* value of less than 0.05 was considered statistically significant. All the statistical analyses were performed using SPSS 21.0 (IBM, Armonk, USA).

## 3. Results

### 3.1. Patients' Characteristics

As Table 1 shows, 102 PJS patients (including 8 children) with a mean age of 28.7 years (range 13–55 y) were enrolled in our study. The male/female sex ratio was 40/62. Among them, 43 (42.2%) patients had a family history of PJS. Moreover, 82 (80.4%) patients had a previous history of laparotomy. 23 patients had two or more hospitalizations. Regarding the history of cancer, 18 patients were diagnosed with cancer (breast cancer in three, colorectal cancer in three, ovarian cancer in two, lung cancer in two, thyroid cancer in two, cervical cancer in two, duodenal malignancy in two, and gastric cancer in two).

### 3.2. Characteristics of the SBE Procedure

All patients underwent CTE examination before the SBE procedure and confirmed the presence of polyps ≥10 mm. As Table 2 shows, a total of 129 hospitalizations (including 248 for SBE procedures, 126 for oral approach, and 122 for anal approach) were performed in 102 patients. A total of 5390 polyps (diameter ≥10 mm) were resected, including 3616 (67.1%) using an oral approach and 1774 (32.9%) using an anal approach. The mean intubation depth was 248.5 ± 62.4 cm and 180.8 ± 32.0 cm for the oral and anal procedures, respectively. The duration of the anal procedure was 121.8 ± 41.6 minutes (range 70–240 minutes), while the duration of the oral procedure was 133.2 ± 44.5 minutes (range 63–245 minutes). The total enteroscopy rate was 42.4% (50/118). Regarding the characteristics of the procedure in children, a total of 723 polyps were resected, including 429 using an oral approach and 294 using an anal approach. The mean intubation depth was 254.5 ± 35.0 cm and 177.3 ± 26.1 cm for the oral and anal procedures, respectively.

23 patients had two or more hospitalizations. For these patients, as Table 3 shows, the maximum size of the resected polyps via the anal approach during the second hospitalization was significantly smaller than that during the first hospitalization ([2.25 ± 1.29] cm vs [4.26 ± 3.51] cm, *P*=0.032). The median number of resected polyps was 38.0 (oral approach) and 8.0 (anal approach) during the first hospitalization and 27.0 (oral approach) and 4.0 (anal approach) during the second hospitalization. The number of resected polyps had a tendency to decrease, although these results were not statistically significant. The median interval between the first and second hospitalization was 20 months (range 5–34 months). As Table 4 shows, the success rate of total enteroscopy in patients with a history of laparotomy was comparable to that in patients without such history (43.9% vs 35.0%, *P*=0.464). The intubation depth via the oral approach of patients with a history of laparotomy was significantly shorter than that of the patients without a history of laparotomy ([241.6 ± 64.2] cm vs [280.9 ± 40.2] cm, *P*=0.008).

For patients with total enteroscopy, 1 patient presented many large polyps that could not be resected in one hospitalization. Among the remaining patients, all polyps larger than 10 mm were successfully resected. The complete treatment rate in these patients was 98% (49/50). For patients without total enteroscopy, 1 patient underwent surgical operation for a giant (>5 cm) and wide-base polyp, 1 patient presented a polyp which is a suspect of malignant tumor in duodenum and was diagnosed as adenocarcinoma after surgical operation, 3 patients presented residual polyps because the polyps were in areas that could not be reached by SBE. Among the remaining patients, all polyps larger than 10 mm in the examined segment of small bowel were resected successfully. After the therapeutic procedure, the patients without total enteroscopy attended for regular follow-up visits. There was no record of intussusception or obstruction caused by small bowel polyps during the follow-up.

### 3.3. Complications

A total of 10 (7.8%) complications occurred among the 129 hospitalizations. Delayed bleeding occurred in four patients. Two patients underwent conservative therapy and two were managed with endoscopic hemostasis. Perforation occurred in three patients, among whom one received conservative therapy and two underwent surgical operations. Acute pancreatitis occurred in three patients, and all of them were treated conservatively with a favorable outcome.

## 4. Discussion

Our study showed that polyps larger than 10 mm in small bowel can be resected effectively. SBE is effective and safe for resection of small bowel polyps in patients with PJS. Treatment of small bowel polyps in PJS has evolved over the past decades. Before enteroscopy development, patients with PJS often undergo acute or elective operations for elective intestinal resection. Hinds et al. reported that 68% of PJS patients underwent a laparotomy because of obstruction before the age of 18 years [22]. In our study, 80.4% of patients had undergone at least one laparotomy. In addition, the risks of gastrointestinal and non-gastrointestinal malignancies significantly increase in patients with PJS [8, 9]. In our study, 18 (17.6%) patients were diagnosed with cancer (breast cancer in three, colorectal cancer in three, ovarian cancer in two, lung cancer in two, thyroid cancer in two, cervical cancer in two, duodenal malignancy in two, and gastric cancer in two). Therefore, except for surveillance of the small bowel, regular check-up on other organs of these patients may be important.

The procedure time in PJS patients is an important piece of information. The mean procedure time was reported to be 70 minutes–108.5 minutes [13, 14, 17]. In our study, the mean procedure time was 121.8 ± 41.6 minutes and 133.2 ± 44.3 minutes for the anal and oral procedures, respectively. The reason may be that most patients have many large polyps. Success rates of total enteroscopy of DBE were reported to be 40% to 80% [23, 24]. Lipka et al. reported that the success rate of total enteroscopy of DBE (range 18.5%–66%) was higher than that of SBE (range 0%–22%) [25]. A recent meta-analysis revealed that the pooled success rate of total enteroscopy of SBE was 21.9% (range 0%–71.4%) [26]. DBE may have a higher success rate of total enteroscopy. In our study, the success rate of total enteroscopy was 42.4%, probably because of high rate of previous laparotomies in these patients. Intraabdominal adhesions would influence the motion of small bowel within the abdominal cavity, impacting the intubation depth of SBE. Hence, we compared the intubation depth between patients with a history of laparotomy and patients without a history of laparotomy. We found that intubation depth via oral approach of patients with a history of laparotomy was significantly shorter than that of the patients without a history of laparotomy ([241.6 ± 64.2] cm vs [280.9 ± 40.2] cm, *P*=0.008). Wang et al. reported a higher success rate of total enteroscopy in patients with a history of laparotomy than in those without a history of laparotomy (61.5% vs 52.4%) [16]. Sakamoto et al. reported a lower success rate of total enteroscopy in patients with a history of laparotomy than in those without a history of laparotomy (62.5% vs 100%) [14]. In our study, the success rate of total enteroscopy in patients with a history of laparotomy was comparable to that in patients without such a history (43.9% vs 35.0%, *P*=0.464), which may be owing to the reduction of the length of the small bowel. Further studies are needed to evaluate the influence of laparotomy on total enteroscopy.

Small bowel polyps sized ≥15 mm (≥10 mm, if possible) should be resected to prevent intussusception in PJS patients [27, 28]. In this study, for patients with total enteroscopy, the complete treatment rate was 98% (49/50). For patients without total enteroscopy, all polyps larger than 10 mm in the examined segment of small bowel were resected successfully. After the therapeutic procedure, the patients without total enteroscopy attended for regular follow-up visits. They underwent CTE examination every 6 months. Patients with polyps <20 mm continued to undergo regular follow-up visits. Patients with polyps ≥20 mm or rapidly growing polyps were arranged to receive SBE treatment within 1 year. If total enteroscopy is achieved, all visible polyps will be removed. If total enteroscopy is not achieved, surgical intervention will be performed. There was no record of intussusception or obstruction caused by small bowel polyps during the follow-up. SBE is effective for resection of small bowel polyps sized ≥10 mm in patients with PJS.

Sakamoto et al. reported that the complication rate in patients with PJS who underwent therapeutic DBE was 6.8% [14]. Mensink et al. reported that the complication rate of diagnostic and therapeutic DBE was 4.3% [29]. The complication rate of this study was 7.8%. The key point may be that most of SBE procedures involved polypectomies of multiple large polyps, which were technically challenging. After conservative therapy or surgical operations, these patients had favorable final outcomes. Regarding the complication of children, only one perforation occurred among the 8 patients. The complication rate was not increased for children as we reported in this study. However, the SBE procedures in children with PJS are difficult. The key reasons are as follows: (1) narrow intestinal lumen, (2) thinner intestinal wall, and (3) sharper angle of enteroscopy. The sample size of children was small, and the safety of SBE in children should be interpreted with caution.

The major limitation of this study was its retrospective and single-center design. However, due to its large number of patients and polypectomies, it allows us to draw some conclusions regarding efficacy and safety.

## 5. Conclusion

In summary, SBE is effective and safe for resection of small bowel polyps in patients with PJS. More well-designed studies, particularly those focusing on the safety and operation technique of SBE in children, are required.

## Figures and Tables

**Table 1:** Patients' characteristics.

	Overall	Children	Adults
No. of patients	102	8	94
Gender, *n* (%)			
Male	40 (39.2)	3 (37.5)	37 (39.4)
Female	62 (60.8)	5 (62.5)	57 (60.6)
Age at treatment, *y* (mean ± SD) (range)	28.7 ± 8.4 (13–55)	15.5 ± 1.4 (13–17)	30.2 ± 7.5 (18–55)
Family history of PJS, *n* (%)	43 (42.2)	3 (37.5)	40 (42.6)
History of laparotomy, *n* (%)	82 (80.4)	5 (62.5)	77 (81.9)
No history of laparotomy, *n* (%)	20 (19.6)	3 (37.5)	17 (18.1)
History of malignancies, *n* (%)	18 (17.6)	1 (12.5)	17 (18.1)
BMI, kg/m^2^ (mean ± SD) (range)	20.6 ± 3.2 (15.0–39.5)	21.4 ± 3.3 (15.6–25.4)	20.6 ± 3.2 (15.0–39.5)
Two hospitalizations, *n* (%)	23 (22.5)	4 (50.0)	19 (20.2)

PJS, Peutz−Jeghers syndrome; BMI, Body Mass Index.

**Table 2:** Details of SBE.

	Overall	Children	Adults
No. of procedures	248	22	226
*Anal approach*			
Procedures (%)	122 (49.2)	11 (50.0)	111 (49.1)
No. of polyps (≥10 mm)	1774	294	1480
Intubation depth, cm (mean ± SD) (range)	180.8 ± 32.0 (50–250)	177.3 ± 26.1 (150–200)	181.1 ± 32.8 (50–250)
*Oral approach*			
Procedures (%)	126 (50.8)	11 (50.0)	115 (50.9)
No. of polyps (≥10 mm)	3616	429	3187
Intubation depth, cm (mean ± SD) (range)	248.5 ± 62.4 (15–350)	254.5 ± 35.0 (200–300)	247.4 ± 64.7 (15–350)
Total enteroscopy (%)	50 (42.4)	5 (50.0)	45 (41.7)
Complications (%)	10 (7.8)	1 (8.3)	9 (7.7)

SBE, single-balloon enteroscopy.

**Table 3:** Endoscopic findings during the patients' hospitalizations.

	First hospitalization	Second hospitalization	*P* value
*Maximum size of resected polyps of SBE, cm (mean* *±* *SD)*			
Anal approach	4.26 ± 3.51	2.25 ± 1.29	0.032
Oral approach	6.00 ± 3.30	4.22 ± 2.90	0.099
*Number of resected polyps of SBE, median (IQR)*			
Anal approach	8.0 (3.0–23.0)	4.0 (1.5–13.5)	0.487
Oral approach	38.0 (6.0–78.0)	27.0 (4.5–61.5)	0.633

SBE, single-balloon enteroscopy.

**Table 4:** Success rate of total enteroscopy and intubation depth of SBE.

	History of laparotomy	No history of laparotomy	*P* value
*Intubation depth of SBE cm (mean* *±* *SD) (range)*			
Anal approach	179.3 ± 33.72	187.5 ± 22.21	0.190
Oral approach	241.6 ± 64.26	280.9 ± 40.24	0.008
Success rate of total enteroscopy	43.9%	35.0%	0.464

SBE, single-balloon enteroscopy.

## Data Availability

All information of patients in these records remained confidential. Further enquiries can be directed to the corresponding author.

## Abbreviations

SBE: Single-balloon enteroscopy
DBE: Double-balloon enteroscopy
PJS: Peutz−Jeghers syndrome.

## Additional Points

*What is known*? (i) Double-balloon enteroscopy has been reported to be useful for the treatment of small bowel polyps in patients with Peutz−Jeghers syndrome. (ii) The diagnosis and treatment procedures in children with Peutz−Jeghers syndrome are difficult. The evidence in regard to the role of enteroscopy in children with Peutz−Jeghers syndrome is limited. *What is new*? (i) Single-balloon enteroscopy is effective and safe for resection of small bowel polyps in patients with Peutz−Jeghers syndrome. (ii) The intubation depth via oral approach of patients with a history of laparotomy was significantly shorter than that of the patients without a history of laparotomy. Laparotomies should be avoided if at all possible. (iii) The complication rate was not increased for children as we reported in this study. However, the sample size of children was small, and the safety of SBE in children should be interpreted with caution.
